# Domains relating to the everyday impact of hearing loss, as reported by patients or their communication partner(s): protocol for a systematic review

**DOI:** 10.1136/bmjopen-2016-011463

**Published:** 2016-09-19

**Authors:** Venessa Vas, Michael A Akeroyd, Deborah A Hall

**Affiliations:** 1 National Institute for Health Research (NIHR) Nottingham Hearing Biomedical Research Unit, Nottingham, UK; 2 Otology and Hearing Group, Division of Clinical Neuroscience, School of Medicine, University of Nottingham, Nottingham, UK; 3 MRC Institute of Hearing Research, University Park, Nottingham, UK; 4 School of Medicine, University of Nottingham, Nottingham, UK

**Keywords:** QUALITATIVE RESEARCH

## Abstract

**Introduction:**

Hearing loss is a highly prevalent condition that affects around 1 in 6 people in the UK alone. This number is predicted to rise by the year 2031 to a staggering 14.5 million people due to the ageing population of the UK. Currently, the most common intervention for hearing loss is amplification with hearing aid(s) which serve to address the issue of audibility due to hearing loss, but cannot reverse its effects. The consequences of hearing loss are multifaceted, as it is a complex condition that can detrimentally affect various aspects of an individual's life, including communication and personal relationships. The scope of these reported issues is so broad that it calls on the need for patient-centred management plans that are tailored to each patient as well as appropriate measures to assess intervention benefit. It is unclear whether current outcome instruments adequately match what patients report as the most important problems for them.

**Methods and analysis:**

The systematic review aims to capture existing knowledge about patients and their communication partner's perspective on the everyday impact of hearing loss. Methods are defined according to the Preferred Reporting Items for Systematic reviews and Meta-analyses for Protocols (PRISMA-P) 2015.

**Ethics and dissemination:**

No ethical issues are foreseen. Findings will be reported in student's thesis as well as at national and international ENT/audiology conferences and in a peer-reviewed journal.

**Systematic review registration number:**

PROSPERO CRD42015024914.

**Strengths and limitations of this study**

- The protocol poses a clearly formulated question about what patients (and communication partners) consider to be the problems related to hearing loss. A systematic review of these domains will help to inform the potential scope for patient-centred management plans and notably selection of outcome measures to assess intervention efficacy and effectiveness.
- The protocol includes a clear methodology for data collection and synthesis with distinct objectives.
- Limitations include a foreseen language bias where articles will only be included in the review if written or obtainable in English.

## Introduction

### Rationale

In the UK, hearing loss affects about 8 million people.^1^ Currently, the most common intervention for people affected by hearing loss is a hearing aid(s), which is typically guided by psychoacoustic measurements such as pure-tone audiometry and speech audiometry. Among researchers, there is general consensus that hearing loss can have a negative impact on various aspects of an individual's quality of life, which extend beyond auditory impairment.^2^ Therefore, current measures may only partially assess the impact of hearing loss. Some of the consequences of hearing loss include burden on relationships with family, participation in social activities and understanding speech.^3^ Numerous questionnaires have been developed that aim to capture some of the issues hearing loss can impose (eg, The Hearing Handicap Inventory for the Elderly),^4^ as well as those that aim to validate an intervention pathway (The Glasgow Profile of Hearing Aid Benefit).^5^ However, there is conflicting evidence within the field of audiology as to which patient-reported outcome measures adequately addresses all of the concepts of hearing loss.^6^ A ‘domain’ in the context of this review protocol refers to a distinct aspect of hearing loss and that is most relevant for assessment derived from recurring descriptive data extracted on the impact of hearing loss. For example, Scarinci *et al*^7^ reported domains derived from individual symptoms that were identified from semistructured in depth interview data rather than reporting solely the individual symptoms. Owing to the large number and broad scope of symptoms associated with hearing loss, identifying appropriate outcome measures for clinical practice and research is challenging. As a result, there is an inconsistency in choice of self-report questionnaires chosen for research purposes as well as the numerous different types of outcome measures used in research.^6^ ^8^

In addition to the uncertainties regarding which types of outcome measures are to be used, the more fundamental concepts of what aspects of adult functioning with hearing loss are considered important also remain ambiguous.^9^ More recently, there has been new emphasis on the psychosocial dimensions of hearing loss and their significance from the perspective of the patient.^10^ In 2001, the World Health Assembly endorsed the International Classification of Functioning, Disability and Health (ICF) for use as an international standard for describing and measuring health and disability. The ICF offers a model that integrates biological, psychological and social aspects of human functioning.^11^ Researchers have recently used the ICF to develop a ‘core set’ which consists of a comprehensive list of categories that are of particular relevance to a specific condition.^9^ ^12^ This process followed a prescriptive methodology, including gaining various perspectives from those affiliated with hearing loss comprising researchers, experts in the field of hearing loss and patients themselves. The aim was to identify which areas of functioning, disability and environment were considered important, from the perspective of adult persons with hearing loss, using seven open questions. The questions used to elicit information from patients developed to specifically address the different components of the ICF framework. Hence, as Granberg *et al*^10^ explained, any patient-reported symptoms that fell outside of this framework could not be assigned to any ICF category and were excluded from further consideration. This suggests some ambiguity as to how well the ICF framework captures the entire scope of symptoms of hearing loss. Excluded responses were not reported in the publication,^10^ and it is possible that essential symptoms may therefore not be reflected in either the ‘comprehensive’ or the ‘brief’ core sets for hearing loss.

The present systematic review aims to collate the existing knowledge about patient and their communication partner's perspective on the negative consequences of hearing loss on everyday life. This includes studies that have reported individual symptoms from patients and communication partners, as well as studies that have categorised individual symptoms into domains. Communication partners are defined as per Manchaiah *et al*: ‘those with whom the person with hearing impairment communicates with on a regular basis. The term communication partner has been used to refer to the significant others which may include their spouse, siblings, children, friends, relatives, colleagues, and carers’.^13^

The purpose of the systematic review is to develop a list of domains on the impact of hearing loss using data from individuals who have been diagnosed with hearing loss or are in regular contact with someone who has. This objective translates into the primary research question: ‘What are the domains reported by adults with hearing loss and their communication partner(s) considered to be problematic in relation to their hearing loss?’ This would include reported problems with aided and non-aided listening, but not with using listening devices themselves. The domains of symptoms could be used to inform patient-centred management plans, as well as to inform the choice of outcome measures to assess treatment benefit.

### Objectives

#### Primary objective

1. The systematic review will collate independent evidence from studies that have reported what adults with hearing loss and communication partners report as problematic in everyday life.
  After domains relating to the everyday impact of hearing loss have been generated from the data, secondary objectives will:
2. Identify similarities and differences in the evidence collected from patients and their communication partner(s).
3. Compare reported difficulties with existing standardised frameworks for clinical assessment, including the ICF.
4. Investigate the reported symptoms in relation to severity of hearing loss, where hearing status is adequately specified.

Secondary analyses will be conducted only where there is a sufficient number of records for a meaningful synthesis of the data.

### Methods and analysis

Methods are reported according to the Preferred Reporting Items for Systematic Reviews and Meta-analyses for Protocols 2015 (Prisma-P) 2015.^14^ Subheadings correspond to the items outlined in the PRISMA-P checklist.

### Eligibility criteria

#### Study designs

Given the large number of closed-set questionnaires in use, eligible study designs span quantitative, as well as qualitative, methodologies. Eligible studies must have employed questionnaires (closed or open questions), focus groups or interviews (structured, semistructured or unstructured) to elicit the data relating to our primary question. Only interventional, observational and cross-sectional studies will be considered for inclusion. The exclusion criteria are (1) studies that do not report hearing loss as the primary condition of interest, (2) case reports, articles for professional magazines and (3) web-based discussion forums due to their limited scientific value.

#### Participants

Participants eligible for inclusion in the review are men and women who have been diagnosed with mild-to-profound hearing loss (including sensorineural, conductive and mixed hearing loss). Eligible participants will also include communication partner(s) or those whom communicate with an individual on a regular basis. Only articles that include a study sample of participants ≥18 years old will be considered.

#### Intervention

The systematic review will include evidence from any intervention studies that assess and report included data collected at the initial assessment (prior to intervention).

#### Comparison

The systematic review does not assess specific interventions.

#### Settings

There will be no restrictions by type of setting. All settings will be considered, including clinical and academic sites.

#### Language

Articles that are not written in English will be excluded as the research team does not have the resource to support translation.

### Timing

All records to be included in the systematic review will have been published on or after 1982. This date was chosen because Granberg *et al*^6^ identified 16 different standardised patient-reported measures for the evaluation of adults with hearing loss, and, of those, the earliest and most frequently identified was the Hearing Handicap Inventory for the Elderly^4^ which was the questionnaire that was published in 1982. It assesses how an individual perceives the social and emotional consequences of hearing loss.^6^

### Information sources

All published journals will be included where they can be identified through the following electronic databases: PubMed, EMBASE, Web of Knowledge, the Cochrane Central Register of Controlled Trials (CENTRAL) and CINAHL. Conference proceedings will be searched using Cos Conference Papers Index (ProQuest) and Web of Science (Thomas Reuters). Case reports will be excluded from the search due to their low scientific value.

Google Scholar will be searched using the specified search terms through every web page that contains eligible records. We will discontinue the Google Scholar search when one page contains no relevant articles. Records that have not been published in peer-reviewed journals, such as book chapters and conference abstracts, will be considered for inclusion, regardless of publication, in order to avoid publication bias.

Hearing loss associations representing patients (eg, Action on Hearing Loss, the Ear Foundation, the IDA Institute, Hearing Loss Association of America) will also be contacted to enquire about commissioned reports.

### Search strategy

In order to ensure that literature identified is comprehensive and rigorous, the present study comprises three steps outlined by PRISMA: identification of records, screening and eligibility assessment. The search process will identify records and the results will be documented. The search process will be reported in sufficient detail so that it is repeatable. This will include details, the search strategy and the number of records retrieved and excluded. Should any changes to the search process be made, then these will be recorded and explained. All internet database searches and contact with hearing loss associations will also be recorded.

The electronic database search will require ‘hearing’ in the article title or abstract, in conjunction with additional relevant search terms such as keywords or in the title or abstract. The search strategies for each database have been designed to be highly sensitive in order to pull as many potentially relevant records as possible, but not too restrictive that records that could be included are missed. To help structure the search, the Population, Intervention, Comparator and Outcomes elements from PICOS were used.^15^ For each of these components, all possible alternative terms were considered (table 1).

**Table 1:** Matrix of the search terms for PubMed, EMBASE and CINAHL

First category AND	Second category AND	Third category
Hearing	Problem	Patient
	Complain*	Communication partner
	Symptom	Partner
	Impairment	Spouse
	Difficult*	Significant (other)
	Concern*	Family
	Impact	

The search terms for PubMed and EMBASE will be guided by: ‘(hearing) AND (problem OR complain* OR symptom OR impairment OR difficult*OR concern* OR impact OR effect)’ OR ‘(hearing) AND (patient OR communication partner OR partner OR significant OR famil*OR spouse)’.

An example search from PubMed is as follows:

(((((((hearing(Title/Abstract)) AND (‘1982/05/01’(Date—Publication) : ‘3000’(Date—Publication)))) AND (((((((((((((((((problem(Title/Abstract)) OR complain*(Title/Abstract)) OR symptom(Title/Abstract)) OR impairment(Title/Abstract)) OR difficult*(Title/Abstract)) OR concern*(Title/Abstract)) OR impact(Title/Abstract)) NOT child*) NOT p*pediatric) NOT hearing screening) NOT hearing protection) NOT hearing conservation) NOT hearing prevalence) NOT hearing incidence) NOT hidden hearing) NOT ‘hidden’ hearing loss) NOT dementia))) AND Humans(Mesh) AND English(lang) AND adult(MeSH))) OR ((((((hearing(Title/Abstract)) AND (‘1982/05/01’(Date—Publication):‘3000’(Date—Publication)))) AND ((((((((((((((((patient(Title/Abstract)) OR communication partner(Title/Abstract)) OR partner(Title/Abstract)) OR significant other (Title/Abstract)) OR famil*(Title/Abstract)) OR spouse(Title/Abstract)) NOT child*) NOT p*ediatric) NOT hearing screening) NOT hearing protection) NOT hearing conservation) NOT hearing prevalence) NOT hearing incidence) NOT hidden hearing) NOT ‘hidden’ hearing loss) NOT dementia))) AND Humans(Mesh) AND English(lang) AND adult(MeSH)).

The first category is combined with the second category as an ‘AND’ statement and similarly, the first category is combined as an ‘AND’ statement with the third category. Records are filtered by English language, human, date and age categories where possible.

## Study records

### Data management

VV (first author) will be responsible for data management and will be the only member of the research team to have editorial rights. A list of records excluded from the review will be reported together with the reasons for exclusion. Reasons as to why any potentially relevant records have been excluded will be highlighted in the document, rather than all of the research evidence identified. Records that have been included will be assigned an ID code to link each record in the master file with along with the full article text. A system of recording decisions made about each article will be produced in Excel.

The study is part of a PhD project. VV will have overall responsibility for the study and shall oversee all study management. VV will have day-to-day responsibility for the study and study conduct. The study will be overseen by two academic supervisors (DAH and MAA).

### Data selection process

Duplicate records will be removed to avoid biased data synthesis.

The first selection step will consider the title information and determine inclusion according to the PICOS and other eligibility criteria. The second stage of selection will consider the abstract for all potentially relevant records that appear to meet the inclusion criteria or for which there is insufficient information in the title to assure inclusion. Articles with a potentially relevant title, but with no available abstract, will be retained and taken through to full-text screening. The third stage of selection will consider the full text of articles that met inclusion or where there is still uncertainty in eligibility. VV and DAH will independently carry out a screen of the titles and abstracts.

If a disagreement arises between the two reviewers (VV and DAH), then the citation of the record will be retained. During the full article screening stage, the full-text papers of the queried citations will be obtained and checked according to the data extraction criteria. If the article is deemed to be ineligible at this stage, then the reasons for exclusion will be documented. Given the large time period of the search citation (33 years), the lead author of the article will not be contacted by email to seek clarification for any missing data. Missing data will be marked as ‘not stated’. During the abstract screening stage, reviewers will assess the potential eligibility of the records using a screening tool (box 1):

Box 1 **Eligibility criteria for abstract screening**

Eligibility criteria: abstract screening

▸ Research aims and objectives of study are appropriate to addressing question(s) of systematic review

▸ Hearing status

▸ Study sample of participants age ≥18 years old

▸ Abstract available in English

▸ Hearing loss is the primary condition of interest

If it can be determined that the article does not meet the inclusion then it can be excluded straight away, specifically, those that are clearly not relevant and those that address the outcome of interest but fail on another criteria, such as population. For those in this latter category, reason for exclusion will be noted.

### Data collection process

The parallel screening of the article titles and abstracts will assure the reliability of the decision process, and for the decision outcomes to be reproducible. A flow chart showing the number of articles remaining at each stage of the selection process will be created to document the study process. A record of any amendments made to the data extraction will be kept for reference. The data extraction form (box 2) serves to ensure the appropriate information is extracted from the articles for the purpose of the review, thus providing consistency. The form will be piloted by VV and DAH to ensure that it extracts information from the article relevant to the review.

Box 2 **Data extraction requirements**

General information

- Researcher performing data extraction (initials)
- Record number (to uniquely identify study)
- Author (last name, first name)
- Article title
- Year of publication
- Type of publication (eg, journal article, report, conference paper, book chapter)
- Country of origin

Study characteristics

- Study design (eg, treatment studies, observational, other)
- Is hearing the primary condition of interest? (eg, yes, no, unclear)
- Primary method for collecting individual hearing loss symptoms (eg, questionnaire, interview, focus group, other)
- Type of questions (eg, open, closed, open and closed)
- Sample size (total number of participants)
- Theoretical framework (if stated)

Participant characteristics

- Mean age
- Gender
- Setting (eg, academic, clinical)
- Hearing status (including mean audiometric thresholds, description of hearing loss severity, aetiology of hearing loss)
- Domains describing patient-reported symptoms
- Measure (eg, interview, focus group)
- Author examples or patient quotes describing their symptoms
- Perspective (eg, perspective refers to self, perspective refers to communication partner)
- Domains describing communication partner-reported symptoms
- Measure (eg, interview, focus group)
- Author examples or communication partner quotes describing their symptoms
- Perspective (eg, perspective refers to self, perspective refers to communication partner)
- Free-field text (any relevant information, ie, not stated in the article)

### Data items

The data extraction form (box 2) will include a comprehensive list of fields corresponding to the study population, design and relevant study findings. If any information is not reported in the record, then ‘not stated’ will be recorded in the corresponding field.

A list of questions and measures (eg, questionnaires) that were used by the authors to the patients and communication partner(s) to obtain the information will be compiled.

Studies may report a mixture of domains of patient-reported symptoms, authors' examples to explain each domain or quotes given by individual patients or their significant others. All of this information will be extracted in the data extraction form.

### Outcomes and prioritisation

The domains of symptoms reported by patients and their communication partner could be used to inform the choice of outcome measures to assess the consequences of hearing as well as treatment benefit.^13^

### Risk of bias in individual studies

A risk of bias assessment will not be carried out as the scope of the review does not involve investigating the effects of a particular intervention for adults with hearing loss.

### Data synthesis

The purpose of this systematic review is to develop a list of domains on the impact of hearing loss using data given by individuals who have been diagnosed with hearing loss or are in regular contact with someone who has been diagnosed. For all included quantitative and qualitative studies, the data items of interest are equivalent (ie, text descriptors of patient-reported symptoms). For included records that have used closed-set questionnaires to assess the impact of hearing loss, only those domains or items of the questionnaire that have been highlighted by the authors because they reflect experienced symptoms will be extracted. For any included records that explicitly report domains, the authors' terminology will be extracted wherever possible. We will also extract any authors' examples or quotes given by individual patients or their significant others relating to the experience of hearing loss. This information will help us to understand the authors' epistemological frame and hence to interpret their concept of each domain. All included records will be used in a narrative synthesis guided by techniques from meta-ethnography and grounded theory to form domains using the domain terms, as well as being informed by the author examples and participant quotes as described above.^16^ ^17^ Relevant information will be displayed in a summary table to show the main characteristics of the included records such as the domains reported, specific examples of symptoms and the data collection methods from which the domains or individual symptoms were obtained (eg, interviews, focus groups etc). We foresee that there will be some variation in the terminology used by authors to describe the same underlying theoretical construct. Therefore, we will pool together examples and quotes given for all domains by the study authors to inform our interpretation of the domains.

The next step of the synthesis will analyse and compare the findings between the included records. Conditional on the amount of relevant data extracted, emerging relationships between studies investigating the secondary objectives will also be carried out. A table will summarise and display the main findings.

We intend to carry out the following prespecified subgroup analyses:

1. Main findings from included records that contain evidence from the perspective of patients that report the everyday impact of hearing loss will be presented in a summary table, and all reported symptoms will be identified.
2. Main findings from included records of the impact of hearing loss reported from communication partners will be presented in a summary table, and all reported symptoms will be identified.
3. To address the other secondary objectives, we will directly compare the reported domains of hearing loss symptoms from each study population. We will devise a table that presents the reported domains of hearing loss-related symptoms corresponding to hearing loss severity (mild, moderate and severe), and we will compare reported symptoms between subgroups.

We will compile a comprehensive list of the questions that were asked by the researchers involved in the study to obtain information from patients and their communication partner(s). We will also review the questions the authors used to elicit information from both study populations, to determine how well the questions were suitable and open enough to elicit unbiased information. We anticipate that not all articles included in the review will report hearing levels of study participants. Therefore, only data from articles that have reported hearing loss will be included in the secondary question, which will seek to associate particular domains with hearing loss severity.

We expect that most of the included records will report narrative data that would have employed qualitative analysis to identify themes or domains. Where appropriate therefore, a quality analysis will be performed using tools designed for qualitative research; Critical Appraisal Skills Programme (CASP).^18^ This 10-point checklist, as well as the items reported in table 2, will seek to ensure that appropriate methods were clearly described and relevant information reported.

**Table 2:** Quality items checklist

Quality assessment	Count of records
Questionnaires only	
Qualitative and questionnaire	
Reporting of population characteristics (eg, age, hearing loss)	
Reference to a theoretical framework	

### Ethics and dissemination

No ethical issues are foreseen. Reports will be guided by the Preferred Reporting Items for Systematic Reviews and Meta-Analyses (PRISMA-P) 2015. We will employ the following dissemination strategies:

- Study results and discussion will be written up in the student's thesis and made accessible via an institutional repository.
- Results will be widely disseminated at conference by the student and academic supervisors.
- Public and patient engagement (eg, articles written for patient association magazines/newsletters).
- A peer-reviewed journal publication complying with Open Access policy.
